# Supplementary figures and images for: Investigation of reward learning and feedback sensitivity in non-clinical participants with a history of early life stress
Source: PLoS One. 2021 Dec 10;16(12):e0260444. doi: 10.1371/journal.pone.0260444 (PMC8664195; doi:10.1371/journal.pone.0260444)

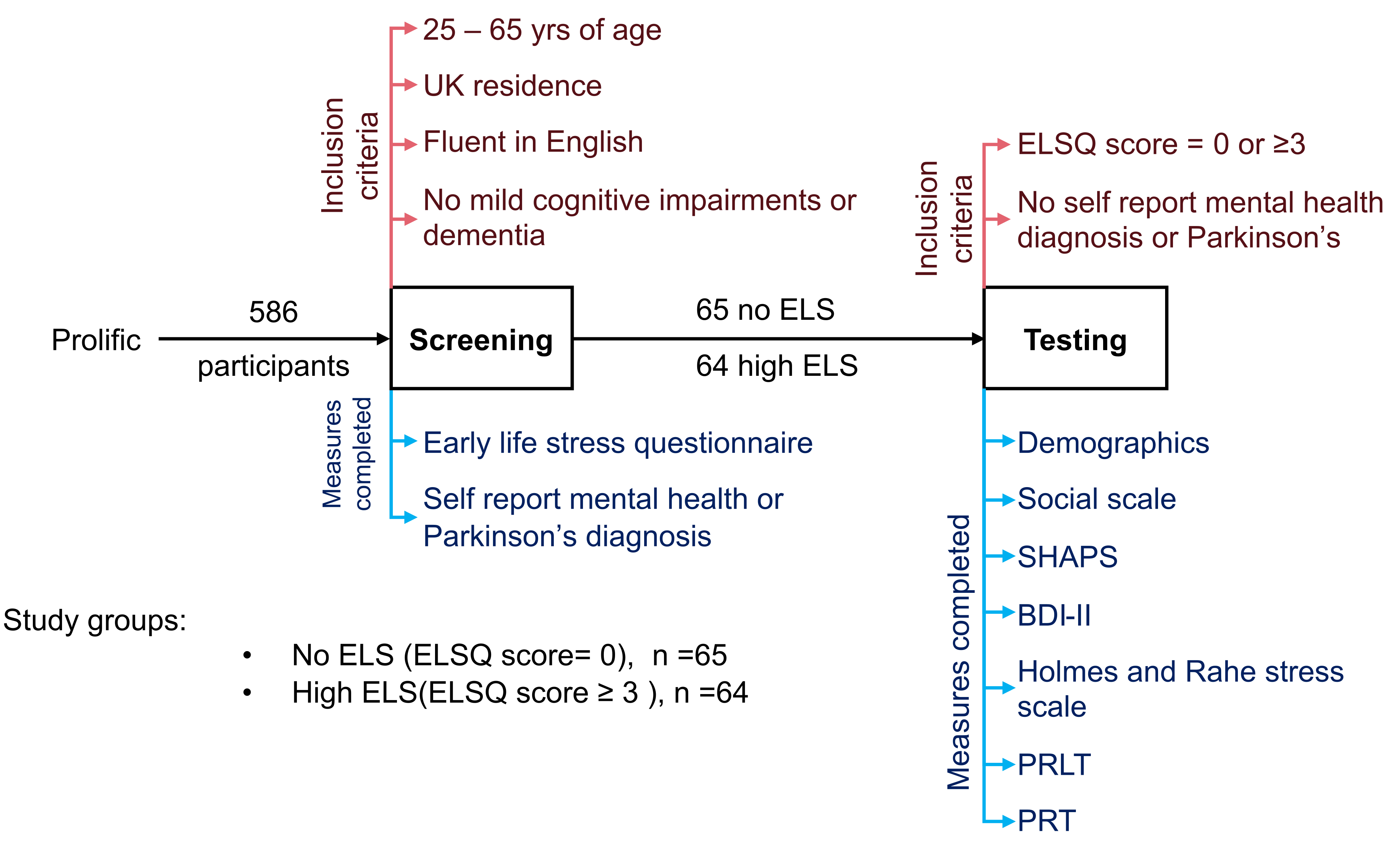

Supplement: S1 Fig — Participants were screened by ELSQ score and then formed into two study groups: no ELS and high ELS. (PNG) [file pone.0260444.s001.png]

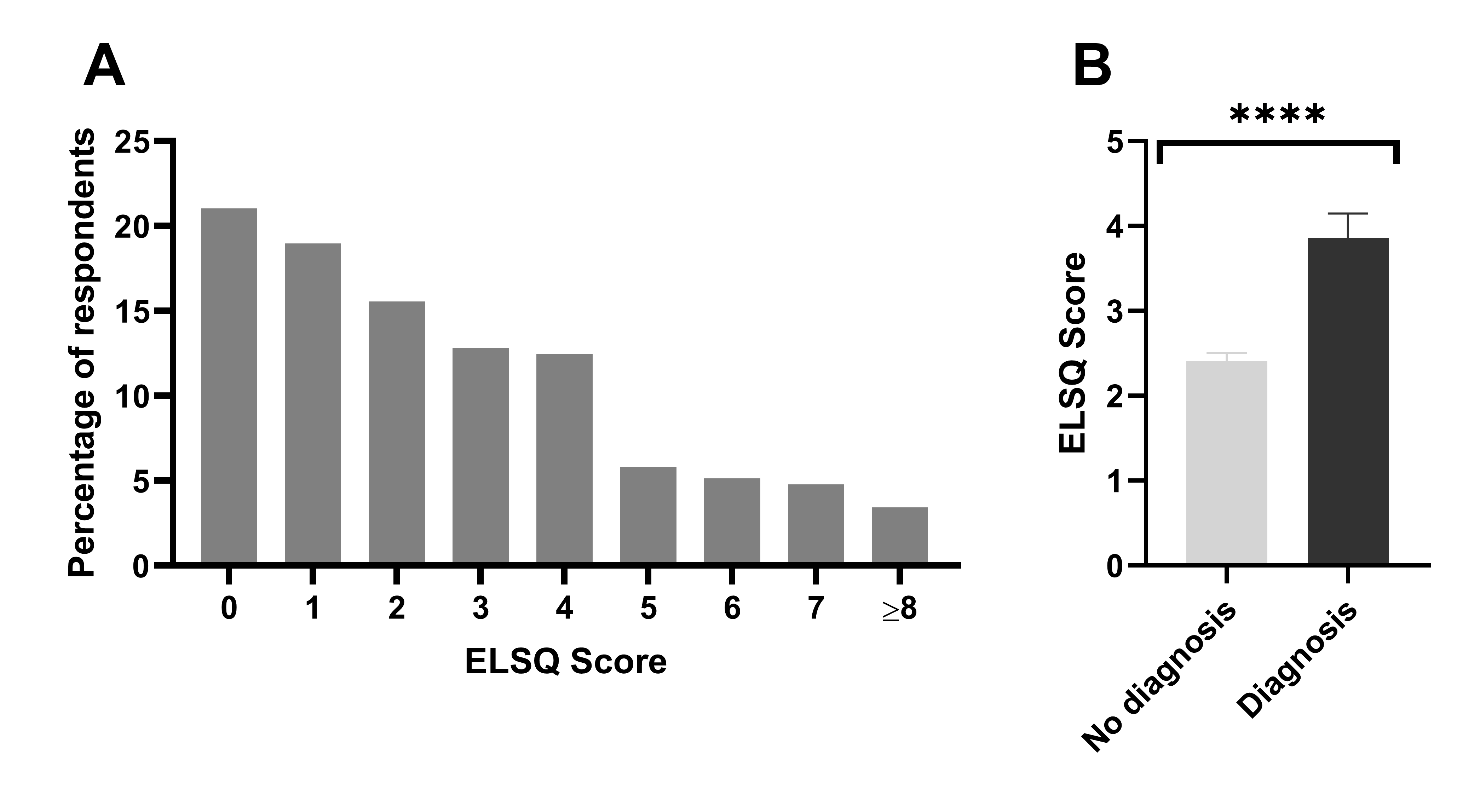

Supplement: S2 Fig — (A) ELSQ scores in the study population. (B) Mental health disorder/Parkinson’s self-report diagnosis by ELSQ score (Mann-Whitney, U = 15725, p < 0.0001). N = 586 participants. (C) ELSQ scores in the study population split by modality of adverse childhood experience. (TIF) [file pone.0260444.s002.tif]

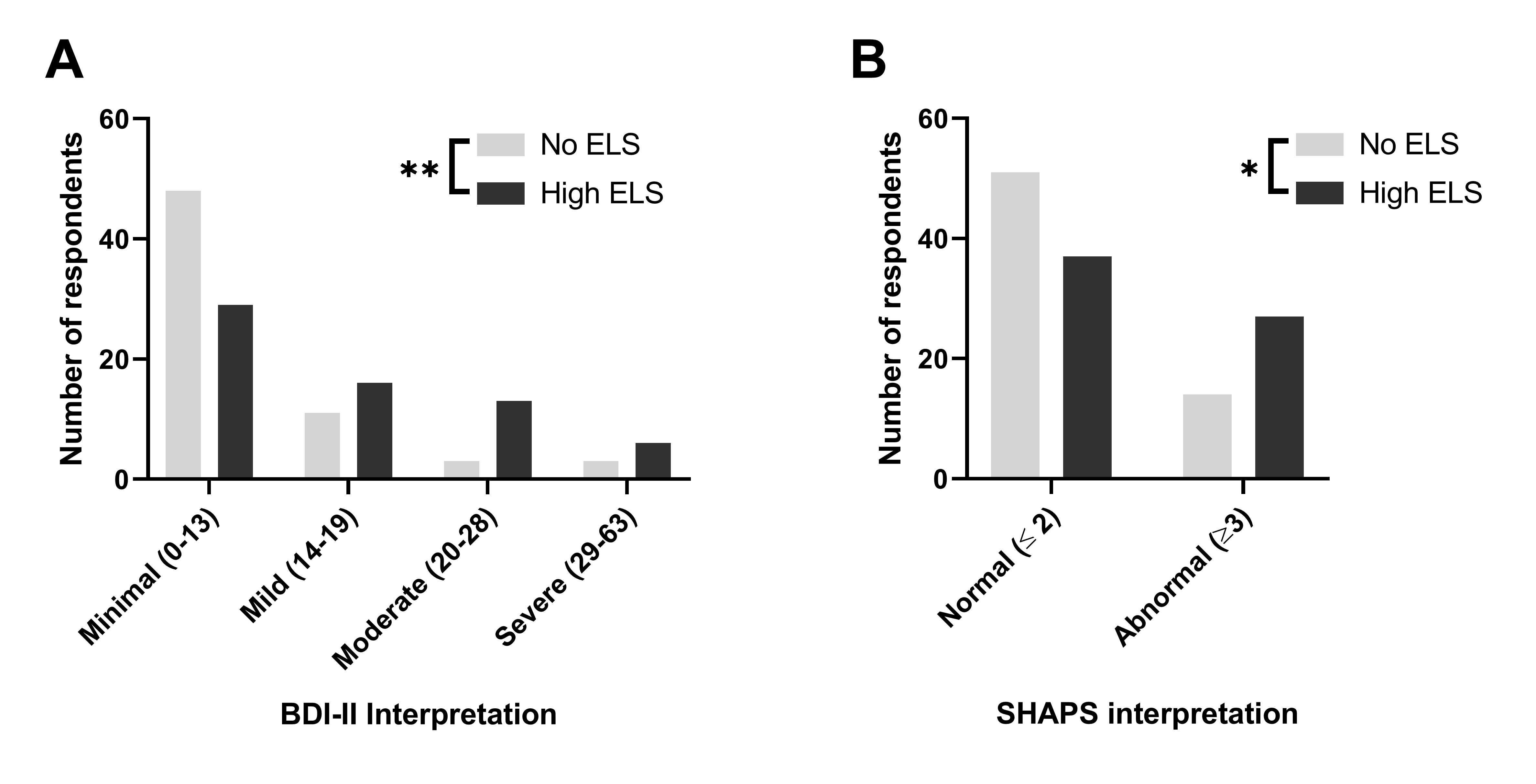

Supplement: S3 Fig — Scores were interpreted following Beck et al., 1996 and Snaith et al., 1995. (A) BDI-II split by severity of depression (chi2, χ2(3) = 12.9, p = 0.005) and (B) SHAPS split by normal or abnormal hedonic responses (chi2, χ2(1) = 6.3, p = 0.012). N = 129 participants (65 no ELS, 64 high ELS). (TIF) [file pone.0260444.s003.tif]

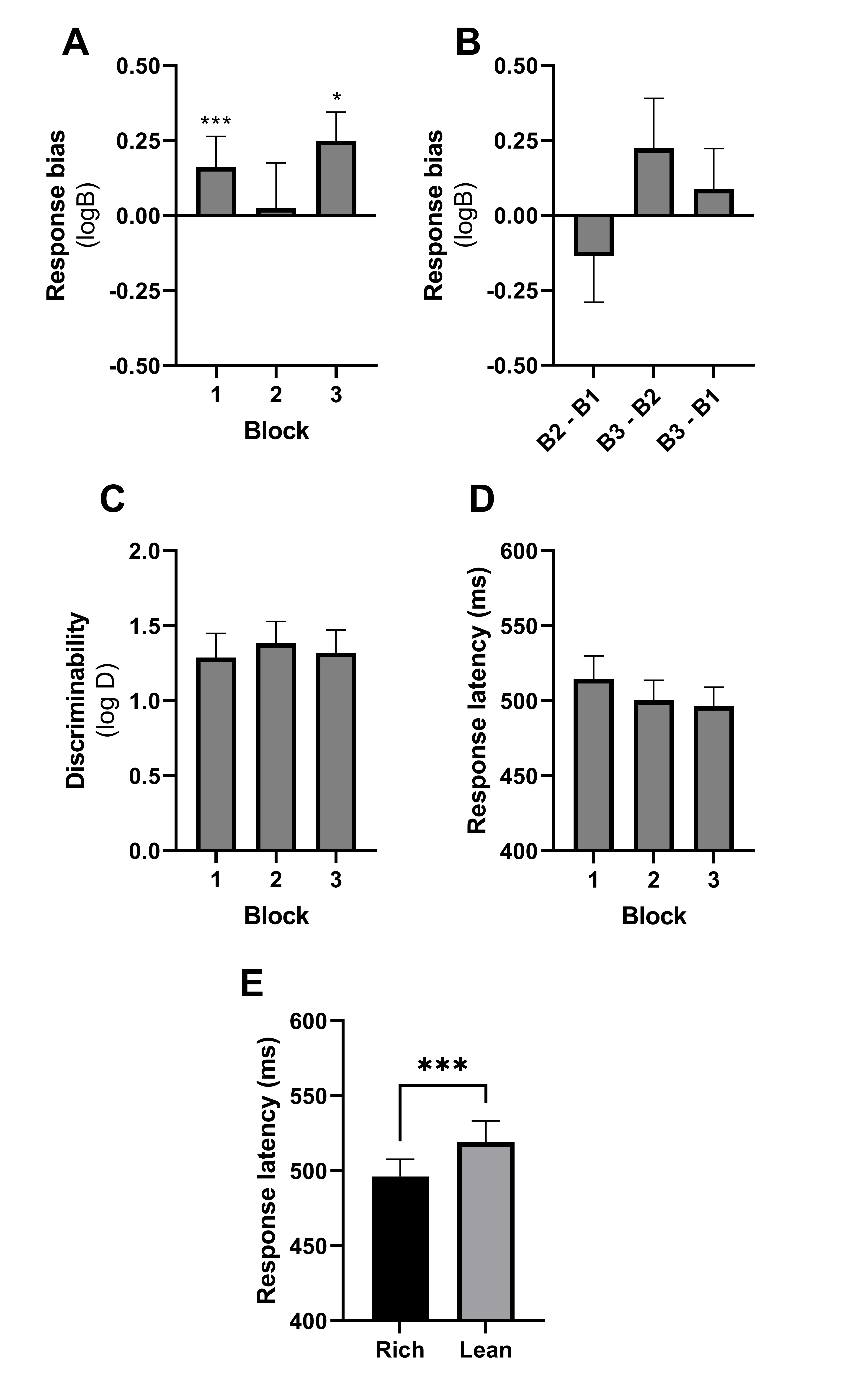

Supplement: S4 Fig — (A) While no overall effect of block was observed, a response bias was observed in blocks 1 and 3 (Wilcoxon signed ranks test, block 1: W = 1087.5, p = 0.001, block 3: W = 916.5, p = 0.038). (B) There was little evidence for response bias strengthening across blocks. Discriminability (C) and response latency (D) did not appear to change over the course of a session. (E) Participants were faster to respond to the rich stimulus than lean (Wilcoxon matched pairs signed ranks test, W = 814, p = 0.0007). N = 56 participants. (TIF) [file pone.0260444.s004.tif]

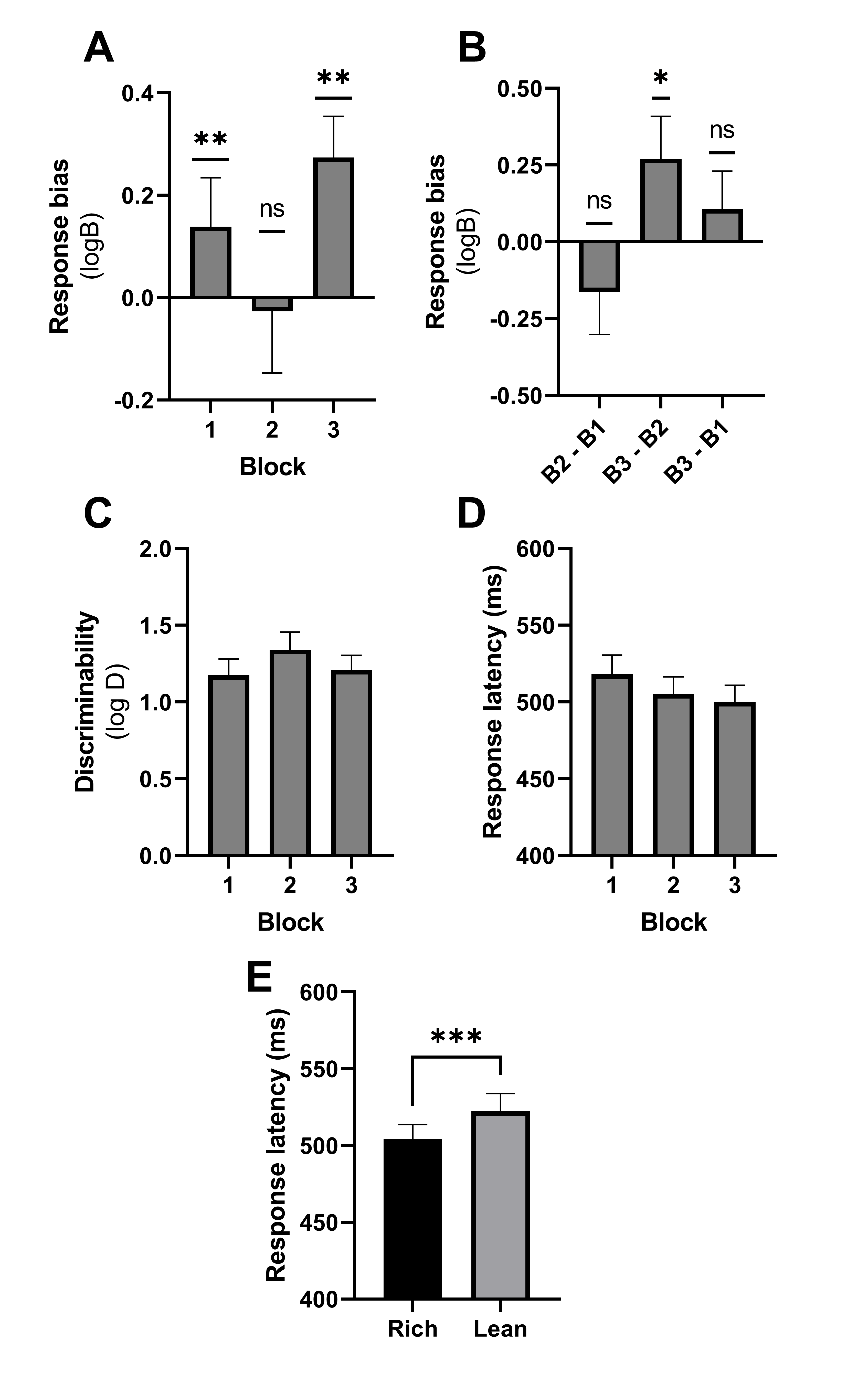

Supplement: S5 Fig — (A) While no overall effect of block was observed, a response bias was observed in blocks 1 and 3 (Wilcoxon signed ranks test, block 1: W = 1174, p = 0.003, block 3: W = 1105, p = 0.004). (B) Response bias strengthened between blocks 3 and 2 (Wilcoxon signed ranks test, W = 873, p = 0.040). Discriminability (C) and response latency (D) did not appear to change over the course of a session. (E) Participants were faster to respond to the rich stimulus than lean (Wilcoxon matched pairs signed ranks test, W = 1372, p < 0.0001). N = 81 participants. (TIF) [file pone.0260444.s005.tif]
