# Supplementary material for: Investigation of reward learning and feedback sensitivity in non-clinical participants with a history of early life stress
Source: PLoS One. 2021 Dec 10;16(12):e0260444. doi: 10.1371/journal.pone.0260444 (PMC8664195; doi:10.1371/journal.pone.0260444)
Supplement: S1 Table — The mean ± standard error are shown for each group with the relevant statistical comparison. (DOCX) [file pone.0260444.s006.docx]

| **Component** | **Explained variance (%)** | **No ELS** | **High ELS** | **Test statistic** | **P value** |
| --- | --- | --- | --- | --- | --- |
| 1 | 94.6 | 4.32 ± 0.24 | 5.65 ± 0.25 | t_127_ = -3.86 | **0.0002** |
| 2 | 3.4 | -0.19 ± 0.21 | 0.20 ± 0.24 | t_127_ = -1.22 | 0.226 |
| 3 | 2.0 | 0.21 ± 0.15 | -0.22 ± 0.18 | t_127_ = 1.79 | 0.076 |

**S5 Table. Principal component analysis of social scale, SHAPS and BDI-II scores.** The mean ± standard error are shown for each group with the relevant statistical comparison.
