# Supplementary material for: Investigation of reward learning and feedback sensitivity in non-clinical participants with a history of early life stress
Source: PLoS One. 2021 Dec 10;16(12):e0260444. doi: 10.1371/journal.pone.0260444 (PMC8664195; doi:10.1371/journal.pone.0260444)
Supplement: S2 Table — (DOCX) [file pone.0260444.s007.docx]

|  | **Principle component** | | |
| --- | --- | --- | --- |
|  | **1** | **2** | **3** |
| Social scale | -0.07 | -0.40 | 0.91 |
| BDI-II | 0.98 | -0.18 | -0.003 |
| SHAPS | 0.17 | 0.90 | 0.40 |

**S6 Table. Principal component analysis component loadings.**
