# Supplementary material for: Investigation of reward learning and feedback sensitivity in non-clinical participants with a history of early life stress
Source: PLoS One. 2021 Dec 10;16(12):e0260444. doi: 10.1371/journal.pone.0260444 (PMC8664195; doi:10.1371/journal.pone.0260444)
Supplement: S3 Table — Data is shown as mean ± standard error and significant p-values are shown in bold. (DOCX) [file pone.0260444.s008.docx]

| **Current trial** | **Previous trial** | **No ELS** | **High ELS** | **Test statistic** | **p** |
| --- | --- | --- | --- | --- | --- |
| Lean | Rich - rewarded | 16.8 ± 2.1 | 18.4 ± 2.2 | U = 1966 | 0.59 |
| Lean | Rich - not rewarded | 16.1 ± 1.8 | 18.6 ± 1.8 | U = 1744 | 0.11 |
| Lean | Lean - rewarded | 19.3 ± 2.1 | 20.5 ± 2.0 | U = 1928 | 0.47 |
| Lean | Lean - not rewarded | 16.8 ± 1.6 | 21.2 ± 1.8 | U = 1928 | 0.097 |
| Rich | Rich - rewarded | 13.1 ± 1.5 | 18.3 ± 2.1 | U = 1697.5 | 0.071 |
| Rich | Rich - not rewarded | 14.2 ± 1.6 | 20.0 ± 2.1 | U = 1597.5 | **0.023** |
| Rich | Lean - rewarded | 13.1 ± 1.4 | 15.5 ± 1.7 | U =1814 | 0.330 |
| Rich | Lean - not rewarded | 14.2 ± 1.4 | 19.6 ± 1.9 | U = 1644.5 | **0.040** |

**S7 Table. Miss-rates, the chance of mis-categorising a stimulus, by previous trial.** Data is shown as mean ± standard error and significant p-values are shown in bold.
