# Supplementary material for: Investigation of reward learning and feedback sensitivity in non-clinical participants with a history of early life stress
Source: PLoS One. 2021 Dec 10;16(12):e0260444. doi: 10.1371/journal.pone.0260444 (PMC8664195; doi:10.1371/journal.pone.0260444)
Supplement: S4 Table — Values are shown for each group as mean ± standard error where appropriate. (DOCX) [file pone.0260444.s009.docx]

| **Measure** | **Control population (n = 56)** |
| --- | --- |
| Sex (% Male) | 51.8 |
| Age (years) | 31 ± 1.1 |
| Employment (% full time) | 48.2 |
| Student status (% student) | 28.6 |
| BDI | 4.1 ± 0.3 |
| SHAPS | 0.30 ± 0.09 |
| SHAPS-C | 20.7 ± 0.6 |

**S10 Table. Demographic and self-report measures in the directly rewarded PRT control population.** Values are shown for each group as mean ± standard error where appropriate.
