# Supplementary material for: Investigation of reward learning and feedback sensitivity in non-clinical participants with a history of early life stress
Source: PLoS One. 2021 Dec 10;16(12):e0260444. doi: 10.1371/journal.pone.0260444 (PMC8664195; doi:10.1371/journal.pone.0260444)
Supplement: S5 Table — Measures by task phase were analysed as multiple Mann-Whitney tests due to non-parametricity of data. #1: Acquisition, Z = -1.38, p = 0.17, First reversal, Z = -2.0, p = 0.045, #2: Acquisition, Z = -1.44, p = 0.15, First reversal, Z = -2.1, p = 0.036, #3: Acquisition, Z = -1.41, p = 0.16, First reversal, Z = -1.2, p = 0.24, #4: Acquisition, Z = -1.95, p = 0.05, First reversal, Z = -0.92, p = 0.36. (DOCX) [file pone.0260444.s010.docx]

| **Task** | **Measure** | **Main analysis** | | | | **Secondary analysis** | | | | | | | |
| --- | --- | --- | --- | --- | --- | --- | --- | --- | --- | --- | --- | --- | --- |
|  |  | **ANOVA / t-test** | | **GLMM** | | **ANCOVA** | | | | **GLMM** | | | |
|  |  | **Test statistic** | **p(group)** | **Test statistic** | **p(group)** | **Test statistic (group)** | **p(group)** | **Test statistic (PCA1)** | **p(PCA1)** | **Test statistic (group)** | **p(group)** | **Test statistic (PCA1)** | **p(PCA1)** |
| PRLT | Rule changes (block) | F_1,127_ = 3.52 | 0.62 | Z = 0.54 | 0.59 | F_1,126_ = 0.054 | 0.82 | F_1,126_ = 0.55 | 0.46 | Z = 0.24 | 0.81 | Z = -0.84 | 0.40 |
|  | Accuracy (block) | F_1,127_ = 0.94 | 0.33 | Z = 0.90 | 0.37 | F_1,126_ = 0.37 | 0.55 | F_1,126_ = 0.94 | 0.34 | Z = 0.54 | 0.59 | Z = -0.99 | 0.32 |
|  | Response latency (block) | F_1,126_ = 5.03 | **0.027** | Z = -2.27 | **0.023** | F_1,125_ = 4.94 | **0.028** | F_1,126_ = 0.12 | 0.73 | Z = -2.26 | **0.024** | Z = -0.34 | 0.73 |
|  | Learning rate | t_127_ = 1.74 | 0.077 | Z = 1.73 | 0.083 | F_1,128_ = 1.59 | 0.21 | F_1,128_ = 1.78 | 0.19 | Z = 1.21 | 0.23 | Z = -1.37 | 0.17 |
|  | Beta | t_125_ = 0.495 | 0.62 | Z = 0.28 | 0.78 | F_1,126_ = 0.009 | 0.93 | F_1,126_ = 2.92 | 0.09 | Z = -0.39 | 0.70 | Z = -2.10 | **0.04** |
|  | Subjective accuracy | t_127_ = 0.065 | 0.95 | Z = -0.02 | 0.99 | F_1,128_ = 0.042 | 0.84 | F_1,128_ = 0.68 | 0.41 | Z = -0.17 | 0.86 | Z = -0.68 | 0.49 |
|  | Win-stay probability (block) | F_1,122_ = 10.4 | **0.002** | Z = 2.77 | **0.005** | F_1,121_ = 6.6 | **0.01** | F_1,121_ = 1.83 | 0.18 | Z = 1.73 | 0.084 | Z = -2.85 | **0.004** |
|  | Win-stay probability (feedback type) | F_1,117_ = 8.6 | **0.004** | Z = 1.84 | 0.066 | F_1,116_ = 5.7 | **0.019** | F_1,121_ = 1.36 | 0.25 | Z = 1.38 | 0.17 | -1.3 | 0.19 |
|  | Lose-shift probability (block) | F_1,126_ = 0.07 | 0.80 | Z = 0.49 | 0.62 | F_1,125_ = 0.004 | 0.95 | F_1,125_ = 0.87 | 0.35 | Z = 0.25 | 0.81 | Z = -0.69 | 0.49 |
|  | Lose-shift probability (feedback type) | F_1,126_ = 0.14 | 0.71 | Z = 1.14 | 0.25 | F_1,125_ = 0.001 | 0.98 | F_1,125_ = 0.98 | 0.32 | Z = 0.89 | 0.37 | Z = -0.68 | 0.50 |
|  | Errors to criterion (task phase) | #1 | #1 | Z = -0.20 | 0.84 | #1 | #1 | #1 | #1 | Z = -0.27 | 0.78 | Z = -0.43 | 0.66 |
|  | Accuracy (task phase) | #2 | #2 | Z = 0.16 | 0.87 | #2 | #2 | #2 | #2 | Z = 0.16 | 0.87 | Z = 0.02 | 0.99 |
|  | Win-stay probability (task phase) | #3 | #3 | Z = 1.11 | 0.27 | #3 | #3 | #3 | #3 | Z = 1.15 | 0.25 | Z = 0.32 | 0.75 |
|  | Lose-shift probability (task phase) | #4 | #4 | Z = 0.15 | 0.89 | #4 | #4 | #4 | #4 | Z = -0.30 | 0.76 | Z = -2.24 | **0.025** |
| PRT | Response bias (block) | F_1,127_ = 0.13 | 0.72 | Z = 0.40 | 0.69 | F_1,126_ = 0.06 | 0.81 | F_1,126_ = 0.10 | 0.75 | Z = 0.29 | 0.77 | Z = -0.15 | 0.88 |
|  | Response bias (block differential) | F_1,127_ = 0.25 | 0.87 | Z = 0.17 | 0.87 | F_1,126_ = 0.08 | 0.78 | F_1,126_ = 0.15 | 0.70 | Z = 0.25 | 0.81 | Z = 0.27 | 0.79 |
|  | Discriminability (block) | F_1,127_ = 4.80 | **0.03** | Z = 2.2 | **0.027** | F_1,126_ = 1.73 | 0.19 | F_1,126_ = 6.08 | **0.015** | Z = 1.33 | 0.18 | Z = -2.49 | **0.012** |
|  | Response latency (block) | F_1,127_ = 0.62 | 0.43 | Z = 0.47 | 0.64 | F_1,126_ = 0.77 | 0.65 | F_1,126_ = 0.77 | 0.38 | Z = 0.21 | 0.84 | Z = -0.74 | 0.46 |
|  | Response latency (stimulus type) | F_1,127_ = 0.60 | 0.44 | Z = 0.55 | 0.58 | F_1,126_ = 0.21 | 0.50 | F_1,126_ = 0.03 | 0.86 | Z = 0.52 | 0.60 | Z = 0.003 | 0.99 |

**S11 Table. Comparison of ANOVA/ANCOVA analytical approach used as main analysis with results obtained using generalised linear mixed models.** Measures by task phase were analysed as multiple Mann-Whitney tests due to non-parametricity of data. #1: Acquisition, Z = -1.38, p = 0.17, First reversal, Z = -2.0, p = 0.045, #2: Acquisition, Z = -1.44, p = 0.15, First reversal, Z = -2.1, p = 0.036, #3: Acquisition, Z = -1.41, p = 0.16, First reversal, Z = -1.2, p = 0.24, #4: Acquisition, Z = -1.95, p = 0.05, First reversal, Z = -0.92, p = 0.36
