# Supplementary material for: Investigation of reward learning and feedback sensitivity in non-clinical participants with a history of early life stress
Source: PLoS One. 2021 Dec 10;16(12):e0260444. doi: 10.1371/journal.pone.0260444 (PMC8664195; doi:10.1371/journal.pone.0260444)
Supplement: S1 File — (DOCX) [file pone.0260444.s011.docx]

**S4 Supporting Information. Supplementary methods for the directly rewarded PRT in a control population.**

An additional cohort of 81 participants were recruited to assess the online PRT using direct monetary compensation.

**Participants**

Eligibility Criteria were: aged 18 – 45 years, fluent in English, resident in the UK, normal or corrected-to-normal vision (self-report), no current or previous diagnosed mental health condition (self-report), reported using a macOS or windows 10 operating system, not taken part in previous prolific studies from the same researcher (i.e., a prolific blocklist was employed) and scored > 65% “correct” on a different reward learning task (the reward learning assay, not discussed here).

**Procedure**

All participants completed a different reward learning task (the reward learning assay) over five consecutive days. On the final day, participants also completed the Probabilistic Reward Task (PRT)[1], Snaith-Hamilton Pleasure Scale (SHAPS)[2] and Beck Depression Inventory (BDI; suicide question removed)[3].

**Probabilistic Reward Task**

The PRT (Pizzagalli et al., 2005) available on the Millisecond test library was employed (using Inquisit v6). The only change made to the task was the monetary amount of reward: participants were informed that they could win up to £5 on this task. Specifically, they were informed that if a correct response is rewarded they will earn four pence.

**Analysis**

Only participants with a minimal BDI score (<13) and normal SHAPS score (≤2) were included in final analysis. The output variables logB and logD were calculated as described elsewhere [1]. Data were both analysed across all blocks for a variable using Friedman tests due to the non-normality of data with response bias data also being compared against a hypothetical mean of zero for each block using Wilcoxon signed rank tests.

**References**

1. Pizzagalli DA, Jahn AL, O’Shea JP. Toward an objective characterization of an anhedonic phenotype: A signal-detection approach. Biol Psychiatry. 2005;57: 319–327. doi:10.1016/j.biopsych.2004.11.026

2. Snaith RP, Hamilton M, Morley S, Humayan A, Hargreaves D, Trigwell P. A scale for the assessment of hedonic tone the Snaith-Hamilton Pleasure Scale. Br J Psychiatry. 1995;167: 99–103. doi:10.1192/bjp.167.1.99

3. Beck AT, Ward CH, Mendelson M, Mock J, Erbaugh J. An inventory for measuring depression. Archives of General Psychiatry. US: American Medical Association; 1961. pp. 561–571. doi:10.1001/archpsyc.1961.01710120031004

4. Beck A, Steer R, Brown G. Beck Depression Inventory. Second Edition. San Antonio, TX; 1996.
